# Epidemic *Achromobacter xylosoxidans* strain among Belgian cystic fibrosis patients and review of literature

**DOI:** 10.1186/s12866-016-0736-1

**Published:** 2016-06-24

**Authors:** Piet Cools, Erwin Ho, Katleen Vranckx, Petra Schelstraete, Bettina Wurth, Hilde Franckx, Greet Ieven, Leen Van Simaey, Sabine Van daele, Stijn Verhulst, Frans De Baets, Mario Vaneechoutte

**Affiliations:** Laboratory Bacteriology Research (LBR), Department of Microbiology, Immunology, and Clinical Chemistry, Faculty of Medicine and Health Sciences, Ghent University, De Pintelaan 185, 9000 Ghent, Belgium; Cystic Fibrosis Centre, Antwerp University Hospital (AUH), Antwerp, Belgium; Applied Maths, Sint-Martens Latem, Belgium; Cystic Fibrosis Centre, Ghent University Hospital (GUH), Ghent, Belgium; Zeepreventorium (Rehabilitation Centre, RHC), De Haan, Belgium; Department of Microbiology, Antwerp University Hospital, Antwerp, Belgium

**Keywords:** *Achromobacter xylosoxidans*, Cystic fibrosis, Epidemic strain, MALDI-TOF MS typing, McRAPD

## Abstract

**Background:**

*Achromobacter xylosoxidans* is increasingly being recognized as an emerging pathogen in cystic fibrosis. Recent severe infections with *A. xylosoxidans* in some of our cystic fibrosis (CF) patients led to a re-evaluation of the epidemiology of CF-associated *A. xylosoxidans* infections in two Belgian reference centres (Antwerp and Ghent). Several of these patients also stayed at the Rehabilitation Centre De Haan (RHC). In total, 59 *A. xylosoxidans* isolates from 31 patients (including 26 CF patients), collected between 2001 and 2014, were studied. We evaluated Matrix Assisted Laser Desorption Ionisation -Time of Flight mass spectrometry (MALDI-TOF) as an alternative for McRAPD typing.

**Results:**

Both typing approaches established the presence of a major cluster, comprising isolates, all from 21 CF patients, including from two patients sampled when staying at the RHC a decade ago. This major cluster was the same as the cluster established already a decade ago at the RHC. A minor cluster consisted of 13 isolates from miscellaneous origin. A further seven isolates, including one from a non-CF patient who had stayed recently at the RHC, were singletons.

**Conclusions:**

Typing results of both methods were similar, indicating transmission of a single clone of *A. xylosoxidans* among several CF patients from at least two reference centres. Isolates of the same clone were already observed at the RHC, a decade ago. It is difficult to establish to what extent the RHC is the source of transmission, because the epidemic strain was already present when the first epidemiological study in the RHC was carried out.

This study also documents the applicability of MALDI-TOF for typing of strains within the species *A. xylosoxidans* and the need to use the dynamic cutoff algorithm of the BioNumerics® software for correct clustering of the fingerprints.

**Electronic supplementary material:**

The online version of this article (doi:10.1186/s12866-016-0736-1) contains supplementary material, which is available to authorized users.

## Background

Non-fermentative Gram-negative bacilli belonging to the genus *Achromobacter* are considered as worldwide emerging bacteria in the cystic fibrosis (CF) population [1], with a predominance of the species *Achromobacter xylosoxidans*. This species can be found in diverse environments, including hospitals [2]. Prevalence in different CF centres varies, ranging from 3 to 30 % of the CF patients being colonized with *A. xylosoxidans* [3–6]. According to the Belgian CF registry, the prevalence of *A. xylosoxidans* in 2013 was 8.9 % in children and 12.2 % in adults.

Factors involved in the emergence of this microorganism remain unknown [7], but are thought to result from selective antimicrobial pressure and the survivor effect, together with improved detection and identification methods.

Also, the clinical relevance of *Achromobacter* colonization remains unclear [8]. In a study by Dunne, Jr. & Maisch [9], the presence of *A. xylosoxidans* was associated with an exacerbation of pulmonary symptoms, but it was difficult to determine the significance of this link because of concomitant isolation of *Pseudomonas aeruginosa*. De Baets et al. [3] pointed to the possible clinical importance of this species in CF, demonstrating that *A. xylosoxidans* infects CF patients with more advanced lung disease without affecting lung function decline. Others demonstrated a lung function decline following infection in a subset of patients with high antibody levels to *A. xylosoxidans* [10]. Several studies indicated an increased need for antibacterial treatment [3, 11]. Otero et al. [12] established that mean annual decline in lung function – measured as annual percentage loss of FEV1 (forced expiratory volume in 1 s) was 2.49 % in nine patients chronically colonized with *A. xylosoxidans* (compared to 1.27 % for intermittently colonized patients) and considered *A. xylosoxidans* as a major pathogen in CF, although caution may be warranted because six of the patients were also colonized with *P. aeruginosa*. Moreover, the level of inflammation caused by *Achromobacter xylosoxidans* was recently shown to be similar to that induced by *Pseudomonas aeruginosa* in chronically infected CF patients [10]. These authors determined cytokine levels in serum and sputum for 11 CF patients colonized by only *A. xylosoxidans* and compared these with those of 21 patients colonized by only *P. aeruginosa*, 17 non-infected CF patients and 11 healthy controls. *A. xylosoxidans* patients were younger, but had a FEV1 decline similar to patients with *P. aeruginosa. A. xylosoxidans* patients had significantly higher sputum TNF-α compared to the other groups of chronically infected patients. The authors concluded that *A. xylosoxidans* can cause a level of inflammation similar to *P. aeruginosa* in chronically infected CF patients and should be considered and treated as a clinically important pathogen in CF.

Little is known about the mode of transmission of *A. xylosoxidans* among CF patients. Current knowledge will be reviewed in more detail in the discussion.

Following some recent severe infections caused by *A. xylosoxidans* among some of our CF patients [13], we decided to determine the epidemiology of CF-associated *A. xylosoxidans* in our two CF centres (Antwerp and Ghent) and we compared the genotypes of the current isolates with those of isolates collected during the period September 2001–October 2002 at the rehabilitation centre in De Haan, where several CF patients from different centres intermittently reside [14].

## Methods

This study was approved by the ethical committee of the Ghent University Hospital (2014/1133). All participants signed informed consent.

### Patients and strains

In total, 59 *A. xylosoxidans* isolates from 31 patients (of which 26 CF patients), collected between 2001 and 2014, were studied (Table 1). Fifty one *A. xylosoxidans* isolates were from 26 CF patients, i.e. 39 isolates from 16 Ghent University Hospital (GUH) patients, eight isolates from 7 Antwerp University Hospital (AUH) CF patients, and four isolates from 3 CF patients collected at the time they stayed at the Rehabilitation Centre at De Haan (RHC), during the period September 2001–October 2002. Furthermore, we included eight epidemiologically unrelated isolates: five isolates were from non-CF patients, i.e. four from patients at the GUH and one from a non-CF patient that had stayed at the RHC recently, and three isolates, including the type strain ATCC 27061^T^, were from culture collections.

**Table 1:** Patients, isolates and typing results

Figure order	Isolate designation	Date isolation (yymmdd)	Stayed at RHC	Co-colonization with *Pseudomonas aeruginosa*	Maldi-TOF Human Interpreter 1	Maldi-TOF Human Interpreters 2&3	Maldi-TOF BioNumerics Dynamic Cutoff Algorithm	McRAPD
25	CF AUH 1	140923	Y	N	B	I	I	1
26	CF AUH 2-1	140923	Y	Y	B	I	I	1
35	CF AUH 2-2	140923	Y	Y	C	I	I	1
34	CF AUH 3	140923	Y	Y	C	I	I	1
2	CF AUH 4	140923	Y	N	A	I	I	1
36	CF AUH 5	140923	Y	Y	C	I	I	1
1	CF AUH 6	140923	N	N	A	I	I	1
3	CF AUH 7	140923	N	Y	A	I	I	1
27	CF GUH 002	020111	Y**	Y	B	I	I	1
45	CF GUH 004-1	120213	Y	Y	E	II	II	2
44	CF GUH 004-2	131015	Y	Y	E	II	II	2
48	CF GUH 004-3	131104	Y	Y	F	II	II	2
43	CF GUH 004-4	131206	Y	Y	E	II	II	2
41	CF GUH 004-5	140303	Y	Y	E	II	II	2
49	CF GUH 004-6	140303	Y	Y	F	II	II	2
57	CF GUH 014-1	131203	Y	Y	P1	P1	P1	P1
56	CF GUH 014-2	140204	Y	Y	P1	P1	P1	P1
6	CF GUH 020	140303	Y	Y	B	I	I	1
42	CF GUH 045	050825	N	Y	E	II	II	2
12	CF GUH 072-1	140328	Y	N	B	I	I	1
29	CF GUH 072-2	140408	Y	N	B	I	I	1
17	CF GUH 072-3	140408	Y	N	B	I	I	1
30	CF GUH 072-4	140429	Y	N	B	I	I	1
40	CF GUH 083	140204	N	N	D	III	I	1
58	CF GUH 086 Duplicate 1	140204	N	N	S3	S3	P2	P2
59	CF GUH 086 Duplicate 2	140204	N	N	S4	S4	P2	P2
15	CF GUH 088-1	131112	Y	Y	B	I	I	1
33	CF GUH 088-2	140106	Y	Y	B	I	I	1
14	CF GUH 088-3	140303	Y	Y	B	I	I	1
22	CF GUH 088-4	140328	Y	Y	B	I	I	1
24	CF GUH 088-5	140414	Y	Y	B	I	I	1
32	CF GUH 098-1	140106	Y	Y	B	I	I	1
18	CF GUH 098-2	140106	Y	Y	B	I	I	1
8	CF GUH 098-3	140328	Y	Y	B	I	I	1
10	CF GUH 098-4	140414	Y	Y	B	I	I	1
38	CF GUH 108-1	140204	Y	N	D	III	I	1
20	CF GUH 108-2	140409	Y	N	B	I	I	1
9	CF GUH 108-3	140414	Y	N	B	I	I	1
39	CF GUH 108-4	140422	Y	N	D	III	I	1
4	CF GUH 135	131104	Y	N	A	I	I	1
5	CF GUH 137	140303	Y	Y	B	I	I	1
13	CF GUH 139-1	140304	Y	Y	B	I	I	1
37	CF GUH 139-2	140303	Y	Y	D	III	I	1
31	CF GUH 172-1	131219	Y	Y	B	I	I	1
16	CF GUH 172-2	140303	Y	Y	B	I	I	1
19	CF GUH 172-3	140328	Y	Y	B	I	I	1
11	CF GUH 172-4	140401	Y	Y	B	I	I	1
7	CF GUH 186	131210	Y	Y	B	I	I	1
21	CF RHC 1 V-1	140407	Y**		B	I	I	1
23	CF RHC 1 V-2	140415	Y**		B	I	I	1
28	CF RHC 2 V	020402	Y*	Y	B	I	I	1
54	CF RHC 3S	010131	Y*	Y	S1	S1	S1	S1
47	NCF Coll 1 Type	Before 1984	N	NA	B	II	II	2
50	NCF Coll 2	Unknown	N	NA	B	II	II	2
53	NCF Coll 3	Unknown	N	NA	B	II	II	2
52	NCF GUH 1	030520	N	NA	B	II	II	2
46	NCF GUH 2	031110	N	NA	B	II	II	2
51	NCF GUH 3	040924	N	NA	B	II	II	2
60	NCF GUH 4	030815	N	NA	S5	S5	S3	S3
55	NCF RHC 1	141204	Y	NA	S2	S2	S2	S2

Isolate designations: *CF* from cystic fibrosis patient, *NCF* from non-cystic fibrosis patient, *AUH* from patient from Antwerp University Hospital, *GUH* from patient from Ghent University Hospital, *RHC* from patient when staying at the Rehabilitation Centre at De Haan (period: 2001–2002), *Coll* from culture collection of Georges Wauters (Université Libre Bruxelles, Belgium), ^*T*^ type strain; −*#* numbering of different isolates from the same patient

Y*: Isolate from CF patient during his/her stay at the RHC, during the period 2001–2002. Affixes ‘S’ and ‘V’ indicate the clusters to which they had been assigned [14]

Y**: Recent isolate from a CF patient of which isolates were also collected during a previous stay (period 2001–2002) at the RHC [14], and for whom the isolates were shown to belong to cluster V

### Species identification by means of *nrdA* gene analysis

The *nrd*A gene, encoding the alpha subunit of the ribonucleoside diphosphate reductase, was amplified using primers nrdA_F (5′-GAACTGGATTCCCGACCTGTTC-3′) and nrdA_R (5′-TTCGATTTGACGTACAAGTTCTGG-3′) [15]. The reactions were performed in a final reaction mixture of 20 μl, containing 10 μl of FastStart PCR Master Mix (Roche Applied Science, Basel, Switzerland), 0.2 μM of each primer, and 2 μl of DNA template. Using a Veriti 96-well thermal cycler (Applied Biosystems, Foster City, US), the following PCR program was run: 94 °C for 5 min, three cycles of 45 s at 94 °C, 2 min at 50 °C, 1 min at 72 °C, and 30 cycles of 20 s at 94 °C, 1 min at 50 °C and 1 min at 72 °C, with a final extension at 72 °C for 7 min. After confirming the presence of amplification products by electrophoresis on 2 % agarose gels, stained with ethidiumbromide, DNA amplicons were purified and sequenced at GATC Biotech (Cologne, Germany) using nrdA_F as sequencing primer.

The obtained *nrd*A gene sequences were compared with the *Achromobacter nrd*A sequences, available at PubMLST: http://pubmlst.org.

### McRAPD for strain typing

DNA was extracted from single colonies by alkaline lysis as described before [16]. RAPD in combination with melting curve analysis of amplified DNA fragments (McRAPD) [17–19] was performed on a Lightcycler 480 (Roche Applied Science). The total reaction mixture per sample was 20 μl and consisted of 2 μl of Lightcycler FastStart DNA Master SYBR Green I (Roche Applied Science), 0.5 mM of primer ERIC2 (5′- AAGTAAGTGACTGGGGTGAGCG-3′), and 2 μl of DNA extract. The PCR protocol started with an activation step of 10 min at 95 °C, followed by 55 cycles of denaturation at 95 °C for 30 s, annealing at 45 °C for 20 s and elongation at 72 °C for 40 s. Melting down of the amplification products started with 1 min at 75 °C followed by increasing the temperature to 97 °C with a ramp rate of 0.1 °C/s. Fluorescence was measured continuously at 530 nm with six acquisitions per °C. The melting curves were calculated using the ‘Tm calling melting curve analysis’ from the LightCycler 480 Software (Roche Applied Science). Interpretation of similarity of these simple patterns was done by visual inspection by two interpreters.

### MALDI-TOF mass spectrometry (MALDI-TOF)

#### Sample preparation

Strains were cultured for exactly 48 h on tryptic soy agar plates + 5 % horse blood (Becton Dickinson, Erembodegem, Belgium) under aerobic conditions at 37 °C. A bacterial suspension of McFarland seven was prepared in 5 ml HPLC-grade water. A total of 300 μl of this suspension was added to an Eppendorf tube containing 900 μl of ethanol 96 %. This suspension was centrifuged at 13,000 × g for 2 min, and the supernatant was discarded. The centrifugation was repeated, and the residual ethanol was carefully aspirated and discarded. The pellet was air dried and resuspended in 20 μl formic acid (70 %), an equal volume of acetonitrile was added and the suspension was centrifuged at 13,000 × g for 2 min. 

#### MALDI-TOF for species identification

One μl cell-extract of each isolate was used for MALDI-TOF MS based identification as described previously [16].

#### MALDI-TOF for strain typing

For each strain, MALDI-TOF spectra were generated of eight spots of one μl-aliquots of cell extract as described previously [20]. Spectra were generated by the FlexAnalysis software (Bruker, Germany) and were exported as text files. These raw spectra (.txt files) were subsequently imported into the BioNumerics® software package 7.5 (Applied Biosystems, Sint-Martens Latem, Belgium).

Raw spectra were processed as follows: the baseline was subtracted using a rolling disc algorithm with a disc size of 50 points, the noise was computed using a Continuous Wavelet Transformation (CWT), and smoothing was performed with a Kaiser digital filter using a moving window with a window size of 20 points and beta of 10 points [21]. A second baseline subtraction was performed using a rolling disc algorithm with a disc size of 200 points, and peaks were detected using a CWT with a signal to noise ratio of 20.

After processing, the eight raw MALDI-TOF mass spectra obtained for each isolate were combined into one composite mass spectrum (main spectrum, MSP) by calculating the average signal intensity for each data point of the eight technical replicates. Raw spectra with a similarity lower than 95 % of the average of the raw spectra were excluded from contribution to the MSP.

### Cluster analysis and multidimensional scaling analysis of MSPs

Two different types of analysis were performed on the MSPs, i.e. cluster analysis based on a similarity matrix and multi-dimensional scaling analysis.

#### Cluster analysis based on a similarity matrix

For the cluster analysis, a similarity matrix was calculated using the curve-based Pearson product-moment correlation coefficient and a similarity-based cluster was constructed using the unweighted pair group method with arithmetic mean (UPGMA) algorithm. In order to try to delimit, by objective means, the relevant clusters from the non-relevant clusters, a dynamic cutoff method (the Cluster Cutoff method of the BioNumerics® software) was used [22].

Briefly, this method draws a virtual line through the dendrogram at a certain similarity level, and from the resulting number of clusters defined by that line, a new, simplified, similarity matrix is created, such that all within-cluster values are 100 %, and all between-cluster values are 0 %. Thereafter, the Point-biserial correlation, i.e. the correlation between this new matrix and the original similarity matrix, is calculated. The level offering the highest Point-biserial correlation is then considered as the one offering groups most supported by the underlying data. As such, the BioNumerics® software allows cutoff values to be different per cluster, i.e. dynamic cutoff (BioNumerics® Manual, available upon request).

The *in silico* defined clusters were subsequently considered as true clusters and subjected to an automated Jackknife test. The Jackknife method determines for each MSP into which of the different defined clusters it matches best by calculating the average or highest similarities of the MSP with each cluster. In cases where a MSP has an equally good match with a member of its own cluster and a member of another cluster, the assignment of the spectrum is spread equally between the two groups. The obtained percentage of correct identifications is a measure of the internal stability of that cluster [23].

#### Multi-dimensional scaling

(MDS) is a dimensionality reduction method, which is a valuable alternative to the dendrogram methods, which often oversimplify the data available in a similarity matrix, and tend to produce overestimated hierarchies. In contrast to dendrogram inferring methods, MDS does not produce hierarchical structures, but instead produces 2D or 3D plots of the similarity matrix, in which the spectra are spread according to their relatedness.

## Results

Species identification by sequencing of the *nrd*A gene indicated that all 59 isolates were *A. xylosoxidans sensu stricto* and not one of the other recently described *Achromobacter* species [15]. Also MALDI-TOF mass spectrometry (MALDI-TOF), which has been shown to be a reliable method for differentiation of the different *Achromobacter* species [24], indicated that all isolates were *A. xylosoxidans sensu stricto*.

**Fig. 1:**
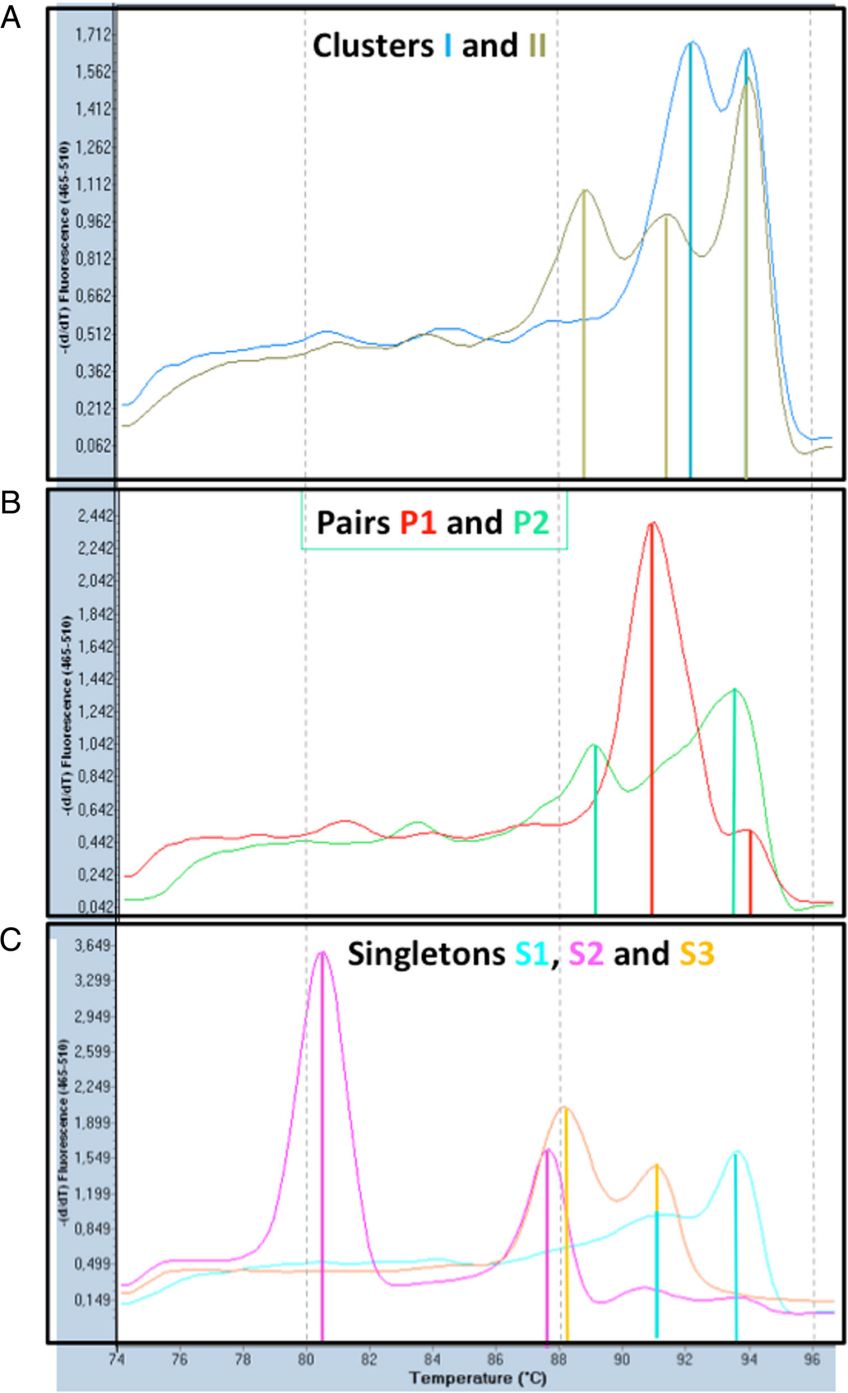
McRAPD profiles, representative for each of the clusters, pairs and singletons. **a** Clusters (I and II); **b**: Pairs (P1 and P2); **c**: Singletons (S1, S2 and S3)

**Fig. 2:**
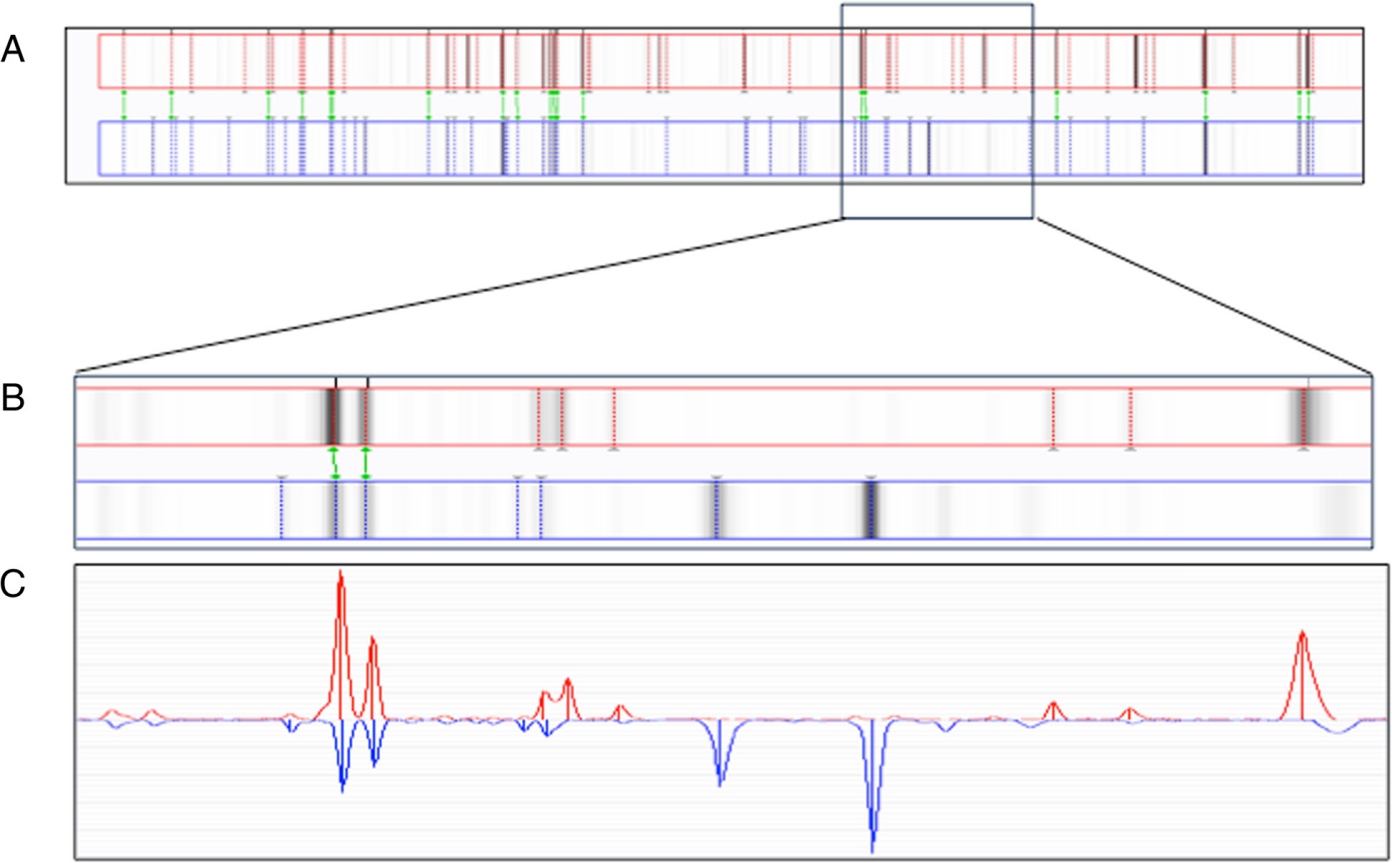
Detailed comparison of two MALDI-TOF MS main spectra (MSPs). **a** Alignment of two MSPs, representative for cluster I (*red*, above) and cluster II (*blue*, below). Spectrum range 2000–10000 m/z. **b**–**c** Enlargement of part of the spectrum (6000–7500 m/z): Gel view representation (**b**) and peak view presentation (**c**). Only peaks with a central line are taken into account for comparison by the BioNumerics® software

**Fig. 3:**
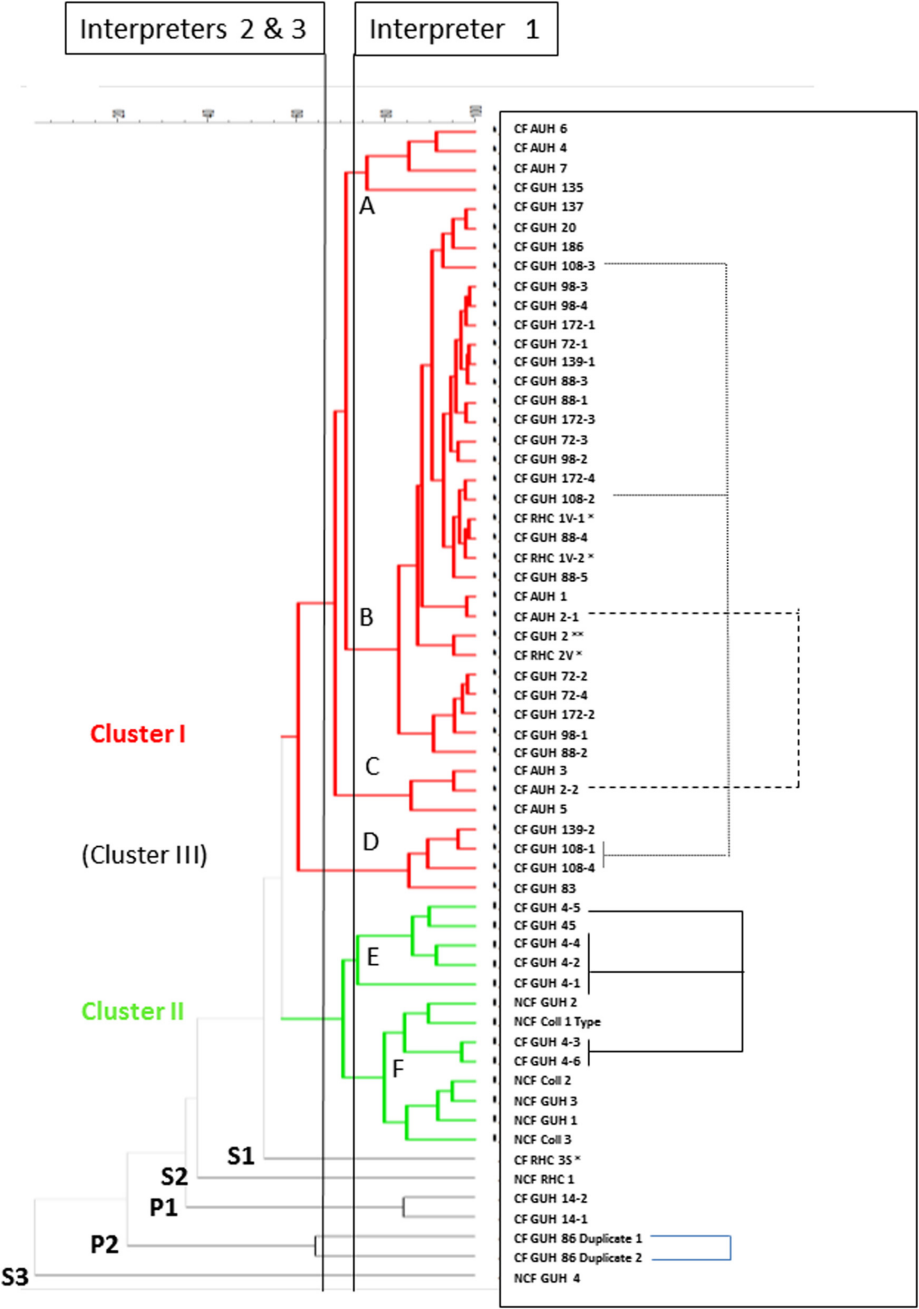
UPGMA constructed tree, based on MALDI-TOF main spectra (MSPs) of 59 isolates (with isolate CF GUH 86–1 tested in duplo). *At the left*: Clusters, Pairs (P) and Singletons (S). Tree lines indicated in bold refer to the clustering obtained by the BioNumerics® software on the basis of MSPs. Isolate designations: *CF* from cystic fibrosis patient, *NCF* from non-cystic fibrosis patient, *AUH* from patient from Antwerp University Hospital, *GUH* from patient from Ghent University Hospital, *RHC* from patient when staying at the Rehabilitation Centre at De Haan (period: 2001–2002), *Coll* from culture collection of Georges Wauters (Université Libre Bruxelles, Belgium), ^*T*^ type strain; −*#* numbering of different isolates from the same patient. Y*: Isolate from CF patient during his/her stay at the RHC, during the period 2001–2002. Affixes ‘S’ and ‘V’ indicate the clusters to which they had been assigned [14]. Y**: Recent isolate from a CF patient of which isolates were also collected during a previous stay (period 2001–2002) at the RHC [14], and for whom the isolates were shown to belong to cluster V. *At the right*: Connector lines indicate some examples whereby multiple isolates of the same patient are clustered differently according to whether the interpretation is done by human observers or by the BioNumerics® software, i.e. patients CF AUH 2, CF GUH 4 and CF GUH 108

Genotyping of the isolates by means of McRAPD indicated the presence of two large groups (clusters I and II) of closely related isolates (Table 1). Fig. 1 represents the different McRAPD profiles that were observed. This result was initially considered unreliable. Although the largest cluster (cluster I) was backed up epidemiologically, as it consisted of isolates that were exclusively from CF patients, cluster II comprised completely unrelated isolates, including the type strain ATCC 27061^T^. Because cluster II was considered to be the result of limited discriminatory power of the McRAPD approach, MALDI-TOF typing was introduced as an independent approach for typing of these isolates. Table 1 presents the MALDI-TOF result for each isolate. Figure 2 illustrates a comparison of the MSPs of two isolates, representative for the two major clusters that were observed. Clustering of the MSPs within the obtained dendrogram (Fig. 3, Additional file 1: Figure S1) was interpreted independently by three human observers and by the dynamic cutoff method, available within the BioNumerics® software. Interestingly, the human observers, using static cut off lines, as indicated by the vertical lines in Fig. 3, concluded that three large clusters (I - III, according to interpreters 2 and 3) or even six clusters (A - F, according to interpreter 1) were present, whereas the dynamic cutoff algorithm indicated the presence of only two clusters (as indicated by the bold tree lines).

Moreover, clustering obtained by means of the dynamic cutoff algorithm was in complete agreement with the McRAPD results, confirming the presence of only two major clusters.

**Fig. 4:**
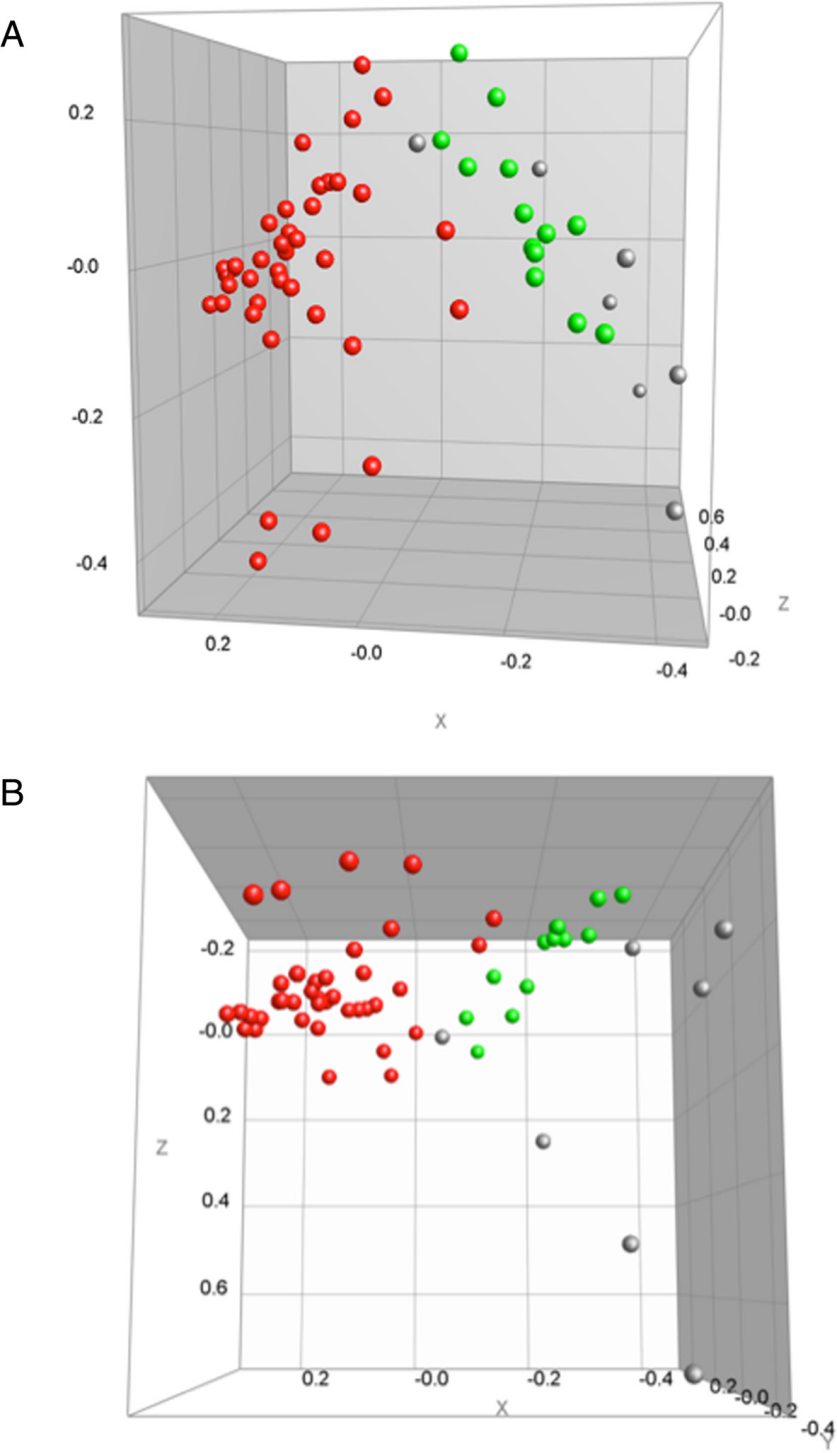
Multi-dimensional scaling (MDS), representing the MALDI-TOF main spectra (MSPs) of the isolates from Cluster I (*red dots*), Cluster II (*green dots*), and the isolates not belonging to one of these clusters (*grey dots*). **a** Side view. **b** Top view

Also MDS analysis (presented in Fig. 4) confirmed independently the presence of two clusters (cluster I profiles: red dots, cluster II profiles: green dots) and the separate position of the pairs and singletons (grey colored points).

Finally, Jackknife analysis confirmed the presence of only two clusters, because of 100 % correct assignation of the individual MSPs to the clusters.

In summary, both McRAPD and MALDI-TOF typing approaches established the presence of a major cluster (cluster I), comprising isolates from CF patients only (*n* = 21, 12 from GUH of which 11 had stayed at the RHC, seven from the AUH of which five had stayed at the RHC, and from two CF patients sampled when staying at the RHC a decade ago). The isolates collected from the two patients when these were at the RHC during the period 2001–2002 have been shown to belong to a major cluster (with ten patients), established at that time at the RHC [14]. This indicates that the major cluster I, established in this study, is the same as the major cluster established at the RHC already more than a decade ago. A minor cluster (cluster II) consisted of 13 isolates from miscellaneous origin, i.e., three collection strains, seven isolates from two CF patients of which one had stayed at the RHC and three isolates from three non-CF GUH patients. A further two pairs of fingerprints (with one pair containing two isolates of the same patient, P2) and three singletons were observed. The singletons included the non-CF patient that had stayed recently at the RHC (S2) and one non-CF patient of the GUH (S1). One isolate, representative for a smaller cluster of four patients at the RHC during the period 2001–2002, was included in the present study and was found to stand alone (S1).

## Discussion

### Background of the study

We decided to study the current epidemiology of the *A. xylosoxidans* isolates from CF patients at two Belgian reference centres (Antwerp (AUH) and Ghent (GUH)), in response to recent cases of serious infections with *A. xylosoxidans* [3]. More than a decade ago, we established the presence of two clusters of genotypically strongly similar isolates among ten (cluster V at the RHC) and four patients (cluster S at the RHC), who stayed at the RHC during the period September 2001–October 2002 [14]. At that time, *A. xylosoxidans* was still considered as a rather innocuous colonizer of the CF airways. Since then, several publications have caused an increased awareness of the possible clinical importance of this species, as has been briefly addressed in the introduction.

### The source of the epidemic strain

The data from this study indicate that most CF patients from the two centres seem to carry the same strain and that this strain was already colonizing ten patients that stayed at the RHC in the period 2001–2002. The large cluster V of the RHC, comprising ten patients [14], corresponds with cluster I of this study. These data suggest continued transmission of *A. xylosoxidans* between Belgian CF patients, since more than 10 years. These findings and the increasing knowledge of *A. xylosoxidans*-associated morbidity over the following period led to stringent segregation measures in our centres.

With regard to the question of the source of this large and longlasting cluster, a single environmental source seems unlikely. The RHC could have served as a source for infection of several patients from different reference centres with the same clone, but the data do not support this possibility. First, at least two clusters (designated S and V) were present already during the period 2001–2002 among patients during their stay at the RHC. Moreover, some CF patients, i.e. CF GUH 004 and CF GUH 014, who have stayed at the RHC, did not carry the cluster I strain, and some patients, who never stayed at the RHC (CF AUH6, CF AUH7 and CF GUH 083), carried the cluster I strain. A final argument against the RHC as the source for this epidemic strain comes from the non-CF patient who stayed recently at the RHC (NCF RHC1), but who carries another strain.

It is difficult to establish to what extent this epidemic spread is due to patient-to-patient transmission, also because most patients – four out of seven at the AUH and 11 out of 16 at the GUH – were co-colonized with *P. aeruginosa* and therefore have been segregated from each other for different periods.

We carried out intensive screening of the hospital environment in the AUH, also during the stay of patients, but could not isolate *A. xylosoxidans* at any occasion, also not from the aerosol equipment.

### Discriminatory power of McRAPD and genetic diversity of *A. xylosoxidans*

The odd observation that several different isolates from very different sources cannot be discriminated may be due i) to close relatedness of these epidemiologically unrelated isolates, ii) to limited discriminatory power of McRAPD or to iii) limited genetic diversity within this species. However, we assume that neither ii) nor iii) is the case. Limited discriminatory power (ii) of McRAPD cannot be the explanation, because this approach has been shown to be discriminative for *A. xylosoxidans* and other Gram-negatives [19], and especially because a completely independent typing approach, i.e. MALDI-TOF, leads to the same observation of limited diversity. Also limited genetic diversity within this species (iii) does not seem to be a valid explanation, because in this study we established several different genotypes by two independent techniques and because extensive genetic diversity is also supported by e.g. the study of Amoureux et al. [2], who observed 35 macrorestriction types among 50 environmental isolates. Therefore, we conclude that the presence of only two types of *A. xylosoxidans* among the set of isolates from CF patients indicates clonal spread of only two strains of *A. xylosoxidans* strains among these patients.

### Comparison of our results with those of other epidemiological studies

#### Establishment of chronic colonization/Predominance of strains

For several patients, we included different isolates, collected over time spans between 1 (CF GUH 72) and 25 months (CF GUH 4). For all patients, we established the presence of only one genotype, indicating that most patients are colonized by only one predominant strain over longer periods. This is in correspondence with several other studies that have reported persistent colonization of CF patients with *A. xylosoxidans*.

Chronic colonization has been established through the persistence of the same clone/strain, whereby clonality was established using diverse molecular typing methods including macrorestriction analysis (PFGE), amplification of repetitive bacterial sequences (rep-PCR) and randomly amplified polymorphic DNA (RAPD) [2, 6, 25–27].

*Achromobacter* colonization of CF patients may be transient, intermittent or chronic [28]. Initial colization with *A. xylosoxidans* results in chronic colonization in 11 to 30 % of the CF patients [3, 4, 11].

Dunne, Jr. & Maisch [9] reported two CF patients, out of eight, that were persistently colonized with *A. xylosoxidans*, as documented by identical rep-PCR patterns for strains repeatedly cultured from the patients. Vu-Thien et al. [29] and Moissenet et al. [25] reported that eight of the 120 children with CF, who were treated at the Hôpital d’Enfants Armand-Trosseau (Paris) between 1990 and 1995, were persistently colonized with a single strain of *A. xylosoxidans*, as documented by macrorestriction analysis of subsequent isolates. Peltroche-Llacshuanga et al. [30] reported two brothers with CF with persistent airway colonisation with *A. xylosoxidans*. Krzewinski et al. [31] used RAPD to genotype between 13 and 19 *A. xylosoxidans* isolates per patient for 15 patients. Thirteen of the 15 patients had a single genotype identified. The other two patients each had an A-B-A pattern, with a single intervening culture with a markedly different genotype and reversion to the original genotype in subsequent cultures. The overall conclusion was that few patients carried more than one genotype of *A. xylosoxidans* whereas for *Stenotrophomonas maltophilia* and *P. aeruginosa* multiple genotypes per patient were frequently observed. In accordance to Krzewinski et al. [31], we also established – within the Belgian CF population – that 64.5 % of the 76 *P. aeruginosa*-colonized patients carried one, 26.3 % two and 9.2 % three *P. aeruginosa* genotype(s) at the same time [32].

For the patient population at the CF rehabilitation centre, we also found less genotypic diversity among the *A. xylosoxidans* strains observed, compared with the *P. aeruginosa* strains. Only two genotypes were present among the 13 patients colonized with *A. xylosoxidans* [14], and although we genotyped multiple *A. xylosoxidans* colonies per patient, only one patient was found to carry both types, whereas all others carried only a single genotype. This again points to the predominant presence of only a single strain per patient. Kanellopolou et al. [33] recovered 34 *A. xylosoxidans* isolates from sputum samples of nine cystic fibrosis patients at a cystic fibrosis department for adults in Athens, Greece. Isolates that were recovered repeatedly from each patient exhibited identical macrorestriction profiles, indicating that the same strain persisted in the lungs of all nine patients. Magni et al. [26] showed a marked genetic relationship between strains isolated from the same patients at different times and Lambiase et al. [11] found, for six patients, that sequential strains obtained in the study period from the same patient showed identical macrorestriction analysis profiles.

Otero et al. [12] considered nine out of the 18 adult *A. xylosoxidans*-positive patients (mean age 26.6 years, range 18–39 years) as chronically colonized.

Dupont et al. [1] documented intrapatient variability and genome evolution, but this diversity was limited compared to the large interpatient diversity, indicating chronic colonization.

It should be noted that the group of Amoureux et al. [6] did not reach this conclusion of limited genotypic diversity per patient. This may be due to a locally different situation or due to the more discriminatory techniques that were used by these authors. To resolve this discongruence, isolates from different locations should be studied with each other’s typing techniques.

In summary, most of the studies indicate that the same strain of *A. xylosoxidans* can persist in the airways of CF patients and that most patients are colonized by a predominant strain. This observation of limited diversity per patient also enables to establish more easily whether the same strain is present among different patients.

#### Co-colonization with *P. aeruginosa*

Patients colonized by *A. xylosoxidans* are frequently co-colonized by *P. aeruginosa*. The six patients that were followed up over a period of time in the study of Lambiase et al. [11] were all co-colonized with *P. aeruginosa*. Otero et al. [12] reported that ten out of 18 patients with *A. xylosoxidans* were co-colonized by *P. aeruginosa*. Vu-Thien et al. [29] reported that six out of the eight patients were co-colonized with *P. aeruginosa*. In our previous study, we found that 11 of the 13 *A. xylosoxidans* positive patients at the RHC were co-colonized with *P. aeruginosa* [14]. In the present study, 15 out of the 23 CF-patients at the two reference centres were co-colonized. In summary, co-colonization with *P. aeruginosa* is frequently observed.

#### Comparison with epidemiology of *A. xylosoxidans* in CF patients from other countries

The observation of this study that one *A. xylosoxidans* strain can spread among several Belgian CF patients corresponds to transmission reported in most other studies, although epidemic strains have been reported less frequently.

Dunne, Jr. & Maisch [9] found that all strains from eight different patients were distinct between patients.

Moissenet et al. [25] found that two out of eight colonized patients harbored the same strain. The two patients colonized by the same strain had overlapping periods of hospitalization and no common source was identified, pointing to person-to-person spread.

Using macrorestriction analysis, Peltroche-Llacshuanga et al. [30] reported identical isolates for the two brothers with CF with persistent airway colonisation with *A. xylosoxidans*.

Krzewinski et al. [31], using RAPD, found 92 *A. xylosoxidans*-positive CF patients from 46 US centres. There were no cases of shared genotypes among different CF centres. Of the seven centers with multiple *A. xylosoxidans*-positive patients, five sites had patient pairs with shared genotypes. Of these, two pairs were siblings, one pair were friends who were frequently hospitalized at the same time, and two were epidemiologically unrelated.

Kanellopolou et al. [33] recovered *A. xylosoxidans * isolates from sputum samples of 71 CF patients at a CF department for adults in Athens, Greece. Isolates from five of the patients were genetically related.

In our previous study [14], carried out among 13 CF patients, staying at the RHC and from whom *A. xylosoxidans* could be isolated, two clusters were observed. The largest cluster comprised ten patients, which was also the size of the largest cluster of patients carrying *P. aeruginosa* at the RHC at that time.

Raso et al. [4] used macrorestriction analysis to characterize 42 *A. xylosoxidans* isolates obtained over 4 years from the respiratory tract of 22 CF patients from Turin, Italy. The 31 typeable isolates were attributed to eight distinct clusters. Lambiase et al. [11] found 53 patients (17.6 % of the total number of 300 patients studied) that had at least one positive culture for *A. xylosoxidans* and established, using macrorestriction analysis, the presence of seven major clusters – comprising between 3 and 9 strains.

Pereira et al. [34] recovered 122 *A. xylosoxidans* isolates from two Brazilian CF reference centres over a 5-year period from 39 patients. Isolates were genetically heterogeneous between patients, but one genotype was present in 56 % of the patients.

In a small study, with 13 Spanish CF patients, Barrado et al. [35] found one pair of CF patients with genetically identical *A. xylosoxidans* isolates.

Dupont et al. [1] found one pair of patients with related isolates, among 13 patients studied in Montpellier, France.

Amoureux et al. [2, 6] collected, from 2011 to 2012, 339 samples in Dijon’s university hospital, in healthy volunteers’ homes in the Dijon area, and in the outdoor environment in Burgundy (soil, water, mud, and plants). A total of 50 strains of *A. xylosoxidans* were detected in hospital (33 isolates), domestic (nine isolates), and outdoor (eight isolates) samples, mainly in hand washing sinks, showers, and water. Genotypic analysis and *bla*_OXA-114_ sequencing revealed a wide diversity among the isolates, with 35 macrorestriction analysis types and 18 variants of oxacillinases. Interestingly, ten isolates from hospital environment were clonally related to clinical isolates previously recovered from hospitalized patients, and one domestic isolate was identical to one recovered from a CF patient (not at the same house). The strain recovered from a shower in the pneumology department was clonally related to that recovered from the sputum of a CF patient who had been chronically colonized since 1995, but had not previously been hospitalized in that ward.

#### The usefulness of McRAPD and MALDI-TOF for typing of *Achromobacter xylosoxidans* isolates

Our data indicate that McRAPD based genotyping and MALDI-TOF based molecular phenotyping of 59 *A. xylosoxidans* isolates are in perfect correspondence. This confirms the usefulness of McRAPD for genotyping of this and other Gram-negative species [19] and indicates that MALDI-TOF is applicable not only for species identification, but also for molecular typing.

#### The usefulness of the BioNumerics® software for interpretation of the clustering within dendrograms

Interpretation of the clusters within a dendrogram by a human observer is usually subjective and is based on a single static threshold line. Apparently, it can be concluded that the clustering reached by the BioNumerics® dynamic cutoff algorithm is more reliable than the interpretations by the human observers, for several reasons. First, there is the complete agreement with the McRAPD-based clustering, including the odd clustering of a set of epidemiologically unrelated strains within cluster II. Second, several isolates that were collected from the same patients, and that were placed into different clusters by the human interpreters, are clustered together by the dynamic cutoff method (see connector lines to the right of Fig. 3, indicating isolates from the same patient or duplicate fingerprints of the same isolate). Third, regarding the seven fingerprints not belonging to clusters I or II, all three human interpreters concluded that there were five singletons and one pair of related strains (P1), whereas the dynamic cutoff method indicated three singletons and two pairs (P1 and P2), in agreement with the McRAPD clustering. Again, the software seems to reach a more reliable conclusion, also because the two MALDI-TOF profiles, clustered in P2 by the software, were considered as representative for two separate types by all three human observers, whereas they actually represent two technical duplicates of the same strain.

## Conclusions

The clinical importance of *A. xylosoxidans* in CF patients has been recognized only recently. Although there have been previous reports on shared genotypes of *A. xylosoxidans* isolates among CF patients, the reported patient clusters were of rather limited size, except for the recent study from Brasil [34]. Here we report the presence of an epidemic strain, colonizing most of the *A. xylosoxidans*-positive patients in two Belgian CF centres and show that it was present already more than a decade ago among several CF patients attending the Belgian rehabilitation centre at De Haan. As patient-to-patient transmission is the most probable explanation for the spread of this epidemic *A. xylosoxidans* strain, we conclude that segregation measurements are mandatory.

## Abbreviations

AUH, Antwerp University Hospital; CF, cystic fibrosis; GUH, Ghent University Hospital; MALDI-TOF, Matrix Assisted Laser Desorption Ionisation-Time of Flight mass spectrometry; McRAPD, Melting curve analysis of randomly amplified polymorphic DNA; RHC, rehabilitation centre, De Haan, Belgium

## Additional file

Additional file 1: Figure S1. Detailed presentation of MALDI-TOF main spectra (MSPs) of 59 isolates (with isolate CF GUH 86–1 tested in duplo). A: dendrogram based on UPGMA; B: detail of the MaldiTOF MS spectra (2000–4000 m/z); C: cluster 1 (red) and cluster 2 (green); D: similarity matrix based on the Pearson correlation. (PDF 117 kb)
