# Supplementary material for: Epidemic Achromobacter xylosoxidans strain among Belgian cystic fibrosis patients and review of literature
Source: BMC Microbiol. 2016 Jun 24;16:122. doi: 10.1186/s12866-016-0736-1 (PMC4919866; doi:10.1186/s12866-016-0736-1)
Supplement: Additional file 1: Figure S1. — Detailed presentation of MALDI-TOF main spectra (MSPs) of 59 isolates (with isolate CF GUH 86–1 tested in duplo). A: dendrogram based on UPGMA; B: detail of the MaldiTOF MS spectra (2000–4000 m/z); C: cluster 1 (red) and cluster 2 (green); D: similarity matrix based on the Pearson correlation. (PDF 117 kb) [file 12866_2016_736_MOESM1_ESM.pdf]

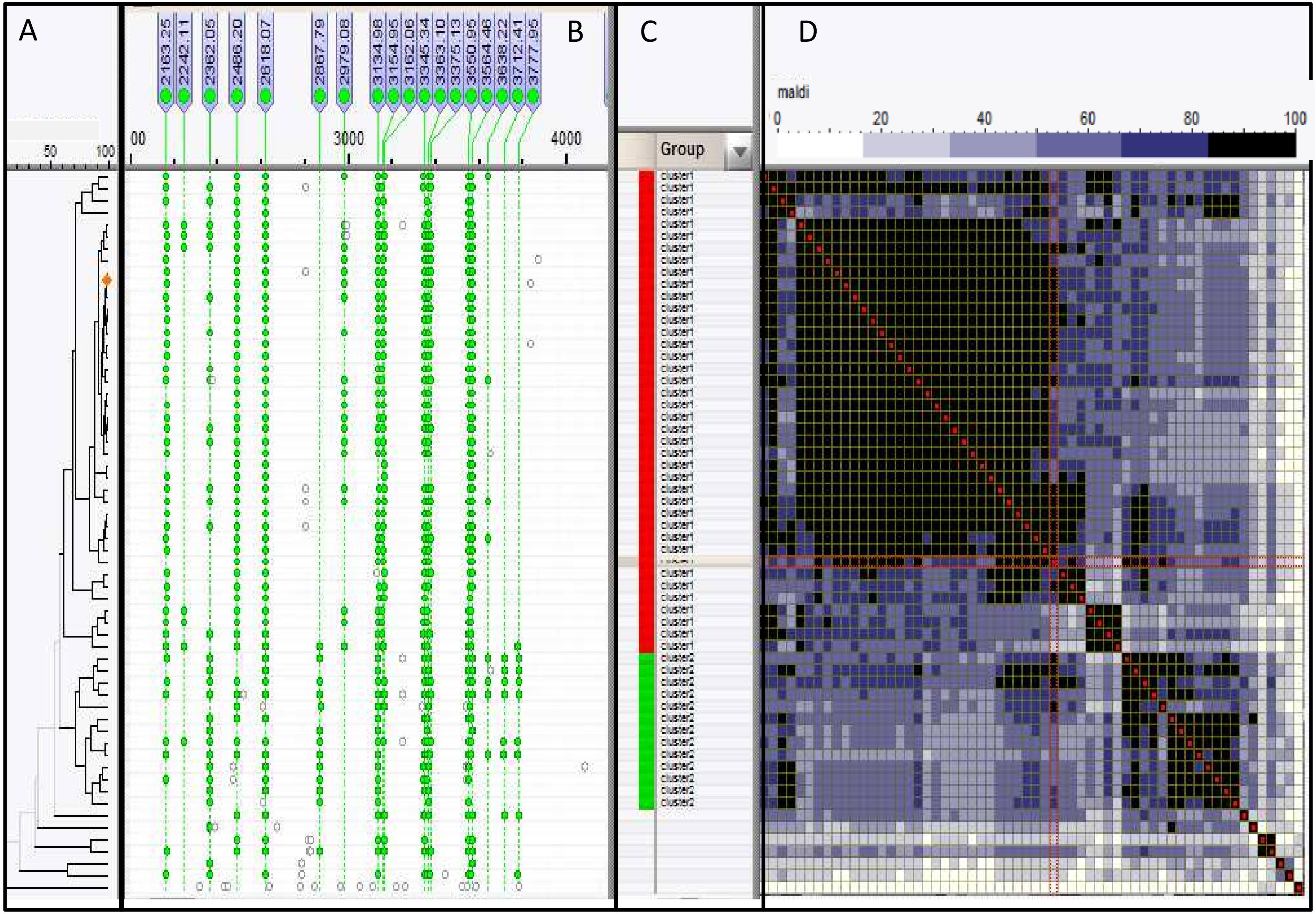

A
B
C
D
2163.25
2242.11
2362.05
2486.20
2618.07
2867.79
2979.08
3134.98
3154.95
3162.06
3345.34
3363.10
3375.13
3550.96
3564.46
3638.22
3712.41
3777.95
50
100
00
3000
4000
Group
cluster1
cluster2
maldi
0
20
40
60
80
100
